# Design and Coupled Moisture–Thermal Transfer Simulation of Opposite Cross-Section Polyethylene Terephthalate Knitted Fabric with Hygroscopic Quick-Drying Capability

**DOI:** 10.3390/ma17174370

**Published:** 2024-09-04

**Authors:** Canyi Lu, Encheng Liu, Yingzhan Li, Guocheng Zhu, Yiqin Shao

**Affiliations:** 1 College of Textile Science and Engineering (International Institute of Silk), Zhejiang Sci-Tech University, Hangzhou 310018, China; lucanyi2027@163.com (C.L.); 2021327100079@mails.zstu.edu.cn (E.L.); yingzhanli@zstu.edu.cn (Y.L.); gchengzhu@zstu.edu.cn (G.Z.); 2 Xiangshan Knitting Research Institute, Zhejiang Sci-Tech University, Xiangshan 315700, China; 3 Zhejiang-Czech Joint Laboratory of Advanced Fiber Materials, Zhejiang Sci-Tech University, Hangzhou 310018, China

**Keywords:** Coolmax, knitted fabrics, moisture absorption and quick drying, moisture–thermal transfer, simulation

## Abstract

In addition to sportswear and outdoor equipment, moisture-absorbent quick-drying fabrics are also widely used in everyday clothing and home textiles. In this study, three types of weft-knitted fabrics were designed using Coolmax fiber and polypropylene fiber. The Coolmax/PP fabric exhibits good stretchability with a strain of 180.5% and achieves a high cumulative individual transfer capability of 691.6%, with a water absorption rate of 50.2%/s. The moisture conductivity gradient presented good moisture and heat conductivity in a simulated human body temperature environment using an infrared camera. Furthermore, mathematical modeling was constructed and visual simulation analysis was conducted to explore moisture–thermal transfer behavior. The simulation results closely align with experimental data, providing insights into designing flexible and wearable quick-drying fabrics for thermal management.

## 1. Introduction

With the continuous improvement of living standards, there is an increasing demand for multifunctional textiles with features such as hygiene, non-ironing, and self-cleaning. From the perspective of sportswear, fibers, yarns, and fabrics impact moisture absorption and perspiration performance [1,2,3]. Fabrics that absorb moisture and dry quickly offer comfort, efficiency, and meet public expectations for clothing comfort [4], environmental protection [5], and health. Knitted fabrics with a dimensional coil structure [6] provide excellent elasticity and breathability [7,8]. Additionally, they offer flexibility and comfort. Polyethylene terephthalate (PET) fiber has the advantage of high tensile strength [9] and elastic modulus [10], excellent thermal stability [11], and good heat and light resistance [12]. Its melting point is about 255 °C, with a glass transition temperature of approximately 70 °C. PET fabrics are known for their durability and washability. However, they suffer from poor moisture absorption. To leverage its strengths and mitigate its weaknesses, it is necessary to modify PET fabric and study the properties of fibers and fabrics after functional treatment. Coolmax fiber is a new type of modified polyester fiber with a large specific surface area [13]. It has a flat cross-section, and the fiber’s longitudinal surface features four grooves that facilitate rapid moisture absorption and transmission away from the skin surface [14], providing excellent moisture management [15]. This results in the fabric having a strong capillary effect [16,17]. Sweat from the skin is swiftly drawn away and transferred to the fabric’s surface for quick evaporation, ensuring dry and comfortable skin. Polypropylene boasts high strength [18], good abrasion resistance, heat resistance, and chemical resistance [19]. Blending Coolmax with polypropylene enhances fabric resilience, abrasion resistance, and imparts a soft full-bodied feel [20].

Numerous studies on moisture-absorbing and quick-drying fabrics have been conducted in order to develop fabrics with superior moisture-absorbing and quick-drying properties. Liu et al. modified polyester (PET) fabrics using a single-sided click chemistry method, significantly enhancing the fabric’s moisture-wicking and fast-drying properties [21] Yu et al. prepared breathable, moisture-absorbent, and antistatic fibrous membranes by incorporating hydrophilic small molecules into PAN through electrostatic spinning [22]. Wang et al. combined a multibranched porous structure with a surface energy gradient to create biomimetic micro- and nanofiber membranes capable of oriented water transport against gravity and rapid drying properties [23].

To conduct comprehensive research on the physical phenomenon of moisture–thermal transfer, numerous domestic and international scholars have engaged in studying fabric models and conducting simulations of moisture and heat transfer. Jin et al. utilized a jacquard weft-knitted fabric simulation algorithm involving three factors to establish and control the fabric model’s effects [24]. Gadeikyte developed a finite element model to analyze the impact of heat transfer characteristics on the wearing comfort of garments [25].

However, in practical situations, the human body generates heat and moisture during exercise. Temperature and moisture interact as they pass through the fabric [26,27,28]. Therefore, investigating the process of heat and humidity transfer in porous fabrics is crucial. To illustrate the changes in moisture and heat within the fabric, a simulation model was established [29]. This model allows for the analysis of moisture diffusion inside the fabric and the distribution of heat [30]. Additionally, it helps to identify the fabric’s rapid moisture absorption characteristics [31]. Moreover, this model supports actual production by enabling fabric evaluation before manufacturing. This ensures timely adjustments to parameters such as blend ratio, thickness, and density, thereby enhancing fabric durability, moisture absorption, and breathability [32], and ultimately leading to greater economic benefits.

Based on the above, the aim of the work is to develop a simulation model of heat and moisture transfer in moisture-absorbing and quick-drying knitted fabrics, and to validate the reliability of the simulated result. Coolmax/propylene knitted fabric was designed and evaluated [33,34], which excels in moisture absorption and wearability. Firstly, the mechanical properties of the fabric were analyzed based on the fabric structure and tensile properties, while the moisture absorption and perspiration performance of the fabrics were analyzed using dynamic moisture transfer methods. Secondly, temperature change characteristics were analyzed under wet conditions simulating human body temperature, and the moisture transfer process was analyzed using an infrared monitoring method to understand the heat and moisture transfer of the fabrics. Finally, based on experimental data, Comsol Multiphysics (6.0) was employed to develop a simulation model for heat and moisture transfer in elastic, moisture-absorbing, and quick-drying knitted fabrics [35]. This work focuses on theoretical research; unlike the single-model simulations conducted by other scholars, it performs coupled moisture–thermal simulations to study the dynamic changes of moisture within the fabric under thermal and humid conditions. This enhances the model’s reliability and comprehensiveness, laying the foundation for future research aimed at optimizing the properties of various application fabrics. The study not only deepens the understanding of the behavior of blended fabrics but also contributes to the development of more effective and efficient textile products.

## 2. Experiments

### 2.1. Preparation of Knitted Fabrics

The fabrics were knitted using a computerized flat knitting machine (SHIMA SEIKI, Wakayama, Japan) with varying proportions of Coolmax/propylene yarns. Raw yarns are fed into the front and back needle beds through the yarn guides. Yarns fed into the front needle bed appear on the fabric’s front side, while those fed into the back needle bed appear on the back side. To ensure that the conclusions are more practical and can effectively guide production practices, we adopted the commonly used fabric blend ratios in the market and adjusted the yarn types to produce three double-layer weft-knitted plain fabrics: Coolmax/PP 50/50 (CP-1), Coolmax/PP 60/40 (CP-2), and Coolmax/PP 65/35 (CP-3). The Coolmax filament was purchased from DuPont (Minneapolis, MN, USA) and has a denier of 75D. The polypropylene (PP) filament was obtained from Senyu International Trading Co. LTD (Dalian, China) and has a denier of 70D.

### 2.2. Characterization

The structure of the knitted fabrics was observed using a Zeiss microscope (Discovery.V20, Carl Zeiss AG, Oberkochen, Germany). The morphologies of Coolmax yarn and PP yarn were characterized using field emission scanning electron microscopy (FESEM, Hitachi S-4800, Hitachi, Tokyo, Japan) at an acceleration voltage of 3 kV. All samples were measured at 20 °C and 65% relative humidity. According to the standard [36], the tensile properties of the fabrics were tested with a tensile testing machine (YG026T-II, Ningbo Textile Instrument Factory, Ningbo, China), with a clamp distance of 50 mm and a crosshead speed of 100 mm/min. Thermal conductivity of the fabric was measured using a HotDisk Thermal Constant Analyzer (Hot Disk AB, Gothenburg, Sweden) according to the standard [37]. Differential scanning calorimetry (DSC) was used to measure the specific heat capacity of the specimens under constant pressure, with a temperature increase rate of 5.00 °C/min and a temperature range from 20 °C to 249.00 °C. The moisture permeability was tested with a computerized fabric hygrometer (YG601-I/II) following the standard [38]. The moisture absorption and quick drying were tested using a liquid moisture management tester (MMT, Sillai Asia-Pacific Ras, Mount Hopkins, AZ, USA) following a standard [39]. Multiple experiments were conducted according to the requirements of different experimental standards to ensure the accuracy and reliability of the results.

### 2.3. Heat and Humidity Conduction Test

To investigate the thermal and humidity comfort of fabrics, a thermal and humidity performance measurement system was developed to simulate human body heat conduction. Compared to standard test methods, the advantage of using an infrared thermal camera to detect the surface temperature of the fabric is that the system can dynamically monitor the change in the surface temperature. Moreover, the surface transition of high and low temperature and water diffusion are displayed on the thermal camera in the process of measurement. Specimens were preconditioned in a constant temperature and humidity laboratory at 20 ± 2 °C and 65 ± 2% humidity for 4 h. A 10 × 10 cm^2^ specimen was then placed on a heated table set at 37 °C. An AFLRI E85 infrared camera (E85, FTIR, Arlington, VA, USA) positioned above the specimen monitored the transfer of water droplets and recorded surface temperature changes at 1 s intervals. Each specimen was monitored for 1 min, with the infrared camera’s temperature range set from 26.9 to 37.0 °C.

### 2.4. Physical Field Modeling

Fabric is a three-dimensional structure composed of many fibers combined through specific arrangement and interweaving techniques. It possesses softness, elasticity, and unique pore characteristics. In the modeling process, the fabric is considered a porous medium material. Herein, Darcy’s Law, phase transfer in porous media, and heat transfer between solid and fluid are selected for comprehensive numerical simulation to analyze the interaction for multiple physical processes.

Water flow through porous fabric involves significant energy dissipation due to frictional effects within the pores, leading to a significant reduction in fluid velocity. Darcy’s Law provides an effective model for this flow phenomenon under low velocity conditions. In this model, water movement is driven by a pressure gradient and is governed primarily by frictional resistance caused by the pore structure. By employing Darcy’s Law, the role of pores within the fabric in water movement was analyzed, and the rate of water transport through the pore structure of the porous medium was calculated using the following formula:

(1) $$\frac{\partial }{\partial t}(\varepsilon_{p}\rho )+\nabla \cdot \rho_{\mu }=Q_{m}$$

(2) $$u=-\frac{\;\kappa }{\mu }\nabla \cdot \rho$$

where *u* is Darcy’s velocity or specific discharge vector (m/s); *κ* is the permeability of the porous medium (m^2^); *µ* is the dynamic viscosity of the fluid (Pa·s); *ρ* is the fluid density (kg/m^3^); $\varepsilon_{p}$ is the porosity; and $Q_{m}$ is the mass source term (kg/m^3^·s).

The Porous Media Phase Transfer interface is used to simulate the transfer of multiple immiscible phases through a porous medium by solving for the average volume fraction of each phase (also known as saturation in the porous medium).

(3) $$\frac{\partial \varepsilon_{p}\rho_{\mathrm{si}}s_{i}}{\partial t}+\nabla \cdot N_{i}=0$$

where $\varepsilon_{p}$ is the porosity of the model material; $\rho_{si}$ is the material density (kg/m^3^); $s_{i}$ is the volume fraction of the phase.

The Heat Transfer in Solids and Fluids interface is used to simulate conductive, convective, and radiative heat transfer. The heat flux q is computed using the formula

(4) $$d_{z}\rho C_{p}\frac{\partial T}{\partial t}+d_{z}\rho C_{p}u\cdot \nabla T+\nabla \cdot q=d_{z}Q+q_{0}+d_{z}Q_{\mathrm{ted}}$$

(5) q=-dk∇T

where ρ is the density of the material (kg/m^2^); $C_{p}$ is the specific heat capacity at constant pressure (J/kg·K); $\frac{\partial T}{\partial t}$ is the rate of temperature change over time, indicating how fast the temperature changes; u is the velocity vector field, indicating the speed and direction of fluid flow; ∇*T* is the temperature gradient, indicating how temperature varies with spatial location; and *q* = *−dk*∇*T* is Fourier’s law of thermal conduction, where *k* is the thermal conductivity.

## 3. Results and Discussion

**Figure 1:**
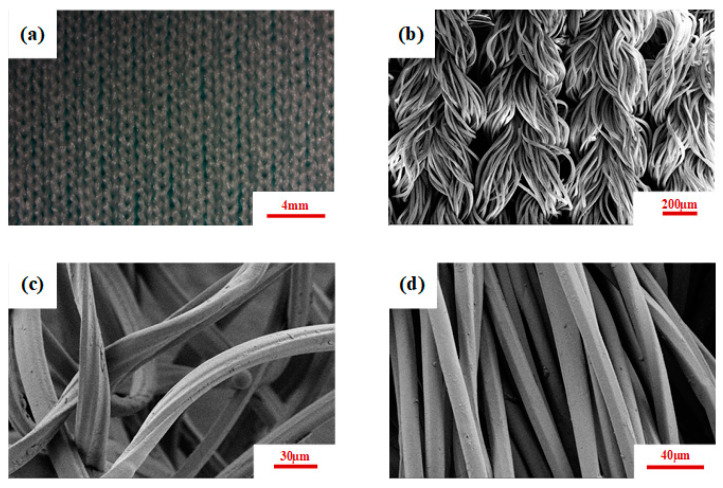
(**a**) The microscopic picture of CP-1 fabric, (**b**) SEM image of fabric, (**c**) Coolmax yarns, and (**d**) Polypropylene yarns.

The real photos of weft-knitted fabrics taken by a Zeiss microscope are shown in Figure 1a. The overall fabric exhibits a smooth surface, clear lines, and fine texture. The SEM image of the fabric is shown in Figure 1b. Figure 1c,d show the SEM images of Coolmax yarns and Polypropylene yarns. Coolmax fiber is polyethylene terephthalate (PET) fiber with a special cross-section. As shown in Figure 1c, the Coolmax fiber has a flat “ten” cross-section, creating a Tetra-Channel structure with four sweat tubes on its surface.

This flat four-groove structure enables adjacent fibers to easily get together, forming many small core pipe-ways with strong capillary effect. This facilitates the rapid discharge of sweat to the fabric surface. Additionally, the specific surface area of the fiber is larger compared to commonly used circular cross-section fibers. Therefore, once sweat is discharged onto the fabric surface, it can quickly evaporate into the surrounding atmosphere. The abnormal cross-section makes a large gap between the fibers, enhancing air permeability. Polypropylene fiber, as shown in Figure 1d exhibits smooth surfaces with good physical properties, such as high strength, wear resistance, and corrosion resistance. Polypropylene fibers were selected to blend with Coolmax to enhance the overall performance.

### 3.1. Moisture Absorption and Quick Drying Performance 

The fabric thickness, density, surface wetting time, water absorption rate, maximum wetting radius, and surface liquid water diffusion rate were tested five times. The results are shown in Table 1. Both fabrics have a thickness of approximately 0.7 mm and density of about 25/cm.

Wetting time refers to the time it takes for the fabric to start absorbing water, identified by the point where the curve depicting water content versus time reaches a slope equal to or greater than tan 15°. According to Table 1, wetting time decreased with an increase in the Coolmax composition ratio. The water absorption rate indicates how quickly the fabric absorbs water per unit time. A higher value reflects stronger moisture absorption capability. Maximum wetting radius refers to the furthest distance water spreads across the fabric surface within a specified time. According to Table 1, it can be seen that CP-3 can reach the fifth level (maximum wetting radius >25 mm), and CP-1 can reach the fourth level (16~25 mm). The liquid water diffusion rate signifies the cumulative transfer speed of liquid water along the radius of the fabric surface during wetting and diffusion to the maximum wetting radius. A higher value indicates better moisture conductivity. The unidirectional transfer index refers to the ability of liquid water to transfer from the surface to the interior of the fabric. Table 1 indicates that all fabrics exhibit good single-direction moisture conductivity. As shown in Figure 1, the cross-section of the Coolmax fiber is a flattened “X” shape, characterized by its cruciform structure and four sweat-wicking channels on the surface, which provides a large specific surface area. The fiber contains numerous fine grooves internally, giving it exceptional capillary action. This unique structure endows Coolmax blended fabrics with outstanding moisture-wicking properties. The research conducted by Raul Fangueiro, which analyzes the influence of Coolmax fiber proportions on moisture management performance [40], aligns with our experimental findings, further validating the reliability of our results.

### 3.2. Tensile Properties

**Figure 2:**
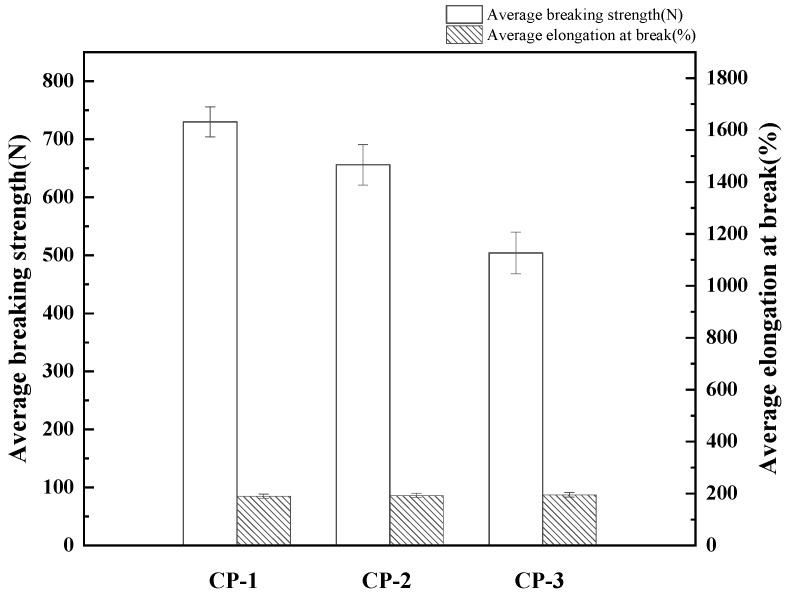
Statistical diagram of tensile property of fabrics.

The tensile properties are displayed in Figure 2. The average breaking strength of CP-1 is 729.94 N, for CP-2 it is 655.9 N, and for CP-3 it is 504.1 N. The average breaking strength of CP-3 is the lowest. However, the average elongation at break of all three samples was close to 190%. In subsequent simulations, two samples with higher breaking strength, CP-1 and CP-2, were used. Irfan et al. investigated the influence of different fibers on the tensile properties of ring-spun yarns and developed an ANN model that accurately predicted these properties [41]. Due to the multi-groove structure of Coolmax fibers, which increases the fiber surface area, moisture removal and evaporation are significantly enhanced. However, this structure also reduces the average breaking strength of Coolmax fibers, thereby impacting the overall tensile performance of the fabric. In blended fabrics, the combination of different fibers influences the final properties of the material. When the Coolmax content is high, it diminishes the good tensile properties of PP, leading to a decrease in the average breaking strength of the fabric.

### 3.3. Heat and Moisture Transfer

First, the samples were pre-treated in a constant temperature and humidity laboratory at 20 ± 2 °C and 65 ± 2% relative humidity for 4 h. A 10 × 10 cm^2^ specimen was cut and covered on a heated table at a temperature of 37 °C. An AFLRI E85 infrared camera on top of the specimen was used to monitor the transfer process of the water droplets and record the temperature change of the surface of the specimen at intervals of 1 s. The overall recording time for each specimen was 1 min.

**Figure 3:**
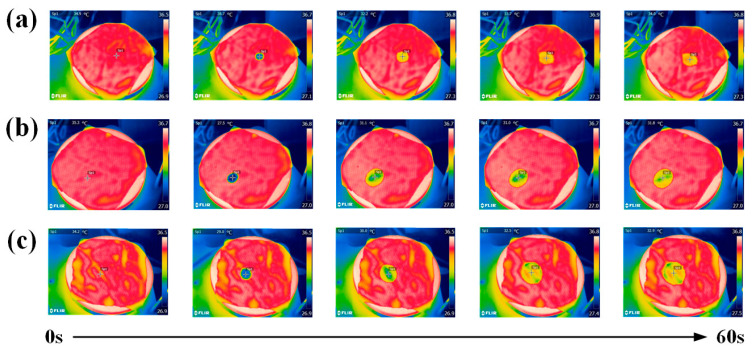
Infrared thermal imaging photos of (**a**) CP-1, (**b**) CP-2, and (**c**) CP-3.

The thermal images of water droplets on the fabric surface are shown in Figure 3, illustrating the heat variations of the droplets on the fabric surface during continuous heating over 60 s. The right column displays the temperature values corresponding to different colors. Water droplets initially formed on the fabric within the first second, then slowly penetrated and diffused through the fabric pores under the influence of gravity and surface tension. The water droplets can expand rapidly within 10 s, indicating that they have good hygroscopic properties. The water droplets diffuse uniformly with rounded edges, as depicted in Figure 3, attributed to the strong capillary action of Coolmax and minimal surface friction, facilitating easy diffusion and infiltration. Moreover, the fabrics form a gradient of moisture conductivity between the upper and lower layers, demonstrating unidirectional moisture transfer.

**Figure 4:**
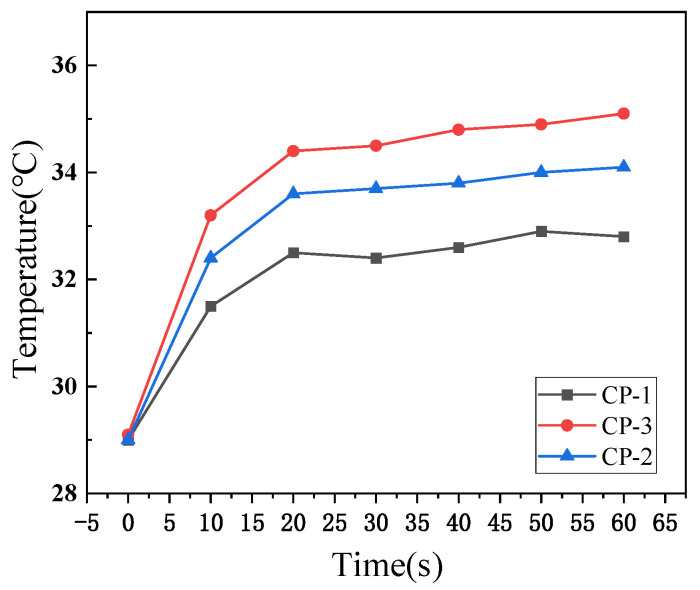
Temperature–time change curve.

The thermal conductivity of fabrics is influenced by fiber type, yarn structure, and fabric structure, forming a multivariate function of structural parameters. Herein, the temperature change of the water droplet on the fabric is shown in Figure 4.

Upon contact with the fabric, the temperature drops to about 29 °C. As the water expands, the temperature gradually increases, reaching its peak in about 30 to 40 s, after which it stabilizes. As the content of Coolmax was increased from 50% to 65%, the trend of the heating rate varied with that of the cooling rate. As shown in Figure 4, when water first contacts the fabric, the temperature rapidly drops to 29 °C. The water diffusion is relatively fast in the initial 10 s, causing the temperature to rise gradually as the water expands. Within about 20 s, the temperature increases rapidly and then stabilizes. This rapid rise is attributed to water’s higher thermal conductivity. Consequently, in a wet environment, the fabric efficiently conducts heat away from the human body.

### 3.4. Coupling Simulation of Moisture and Heat

**Figure 5:**
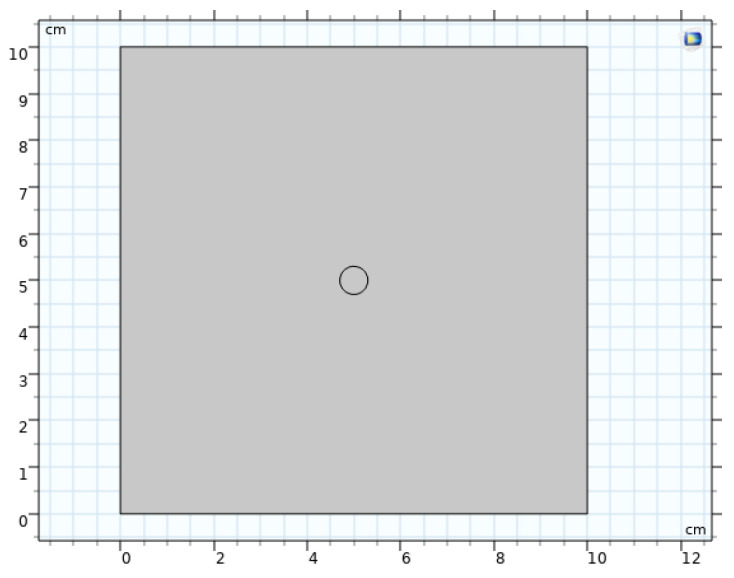
Simulation model of thermal and moisture conduction in fabrics CP-2 and CP-1 with initial water droplet formation and diffusion.

Based on the aforementioned experiments, the thermal and moisture conduction properties of two fabrics, CP-2 and CP-1, were simulated. A square with a side length of 10 cm was designed to simulate the fabric, with a circle of 0.3 cm radius placed at its center to represent the initial size of a water droplet upon dripping onto the fabric (Figure 5). When the water first drops on the surface, surface tension causes it to form a droplet. Then the fabric’s porous structure and the core suction effect causes the water to diffuse within the fabric. 

For simulation purposes, the fabric interior is assumed to be homogeneous, with uniform thermal properties throughout and adiabatic boundaries. Therefore, in the simulation, moisture diffuses towards the fabric surface along the periphery of the circle, where the circle area represents the initial diffusion area of the water droplet. The fabric parameters involved in the numerical simulation of this paper are shown in Table 2.

**Figure 6:**
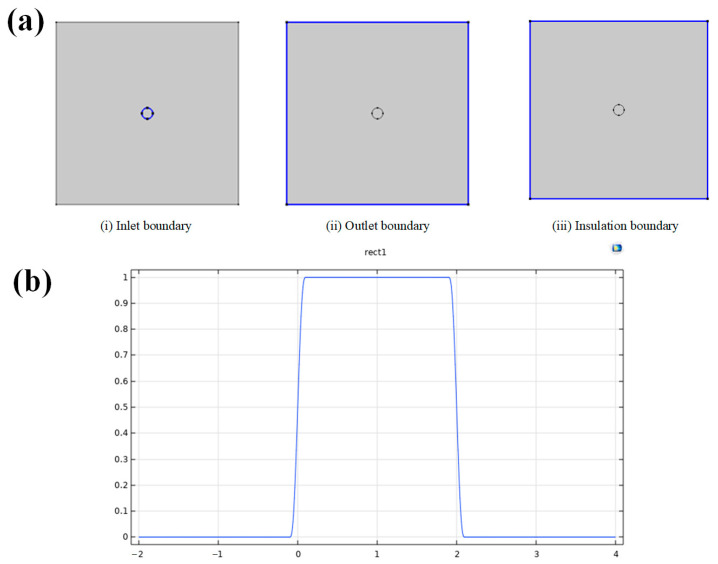
(**a**) Boundary condition setting of fabric. (**b**) Rectangular wave function plot.

Based on the design of the fabric’s heat and moisture transfer equivalent model, Figure 6a illustrates the boundary settings for moisture input and output, as well as the insulation boundaries of the fabric. In the porous media phase transfer, water is supplied to the fabric models using a mass flux method. The time of moisture supply is controlled using a rectangular wave function, depicted in Figure 6b, where the control time shown is 2 s. To ensure smooth operation, a 0.2 s transition zone was added to the rectangular wave function to mitigate abrupt changes, resulting in a gradual slope at the 2 s mark in the graph. This treatment enhances the convergence of the calculation without affecting the simulation results.

**Figure 7:**
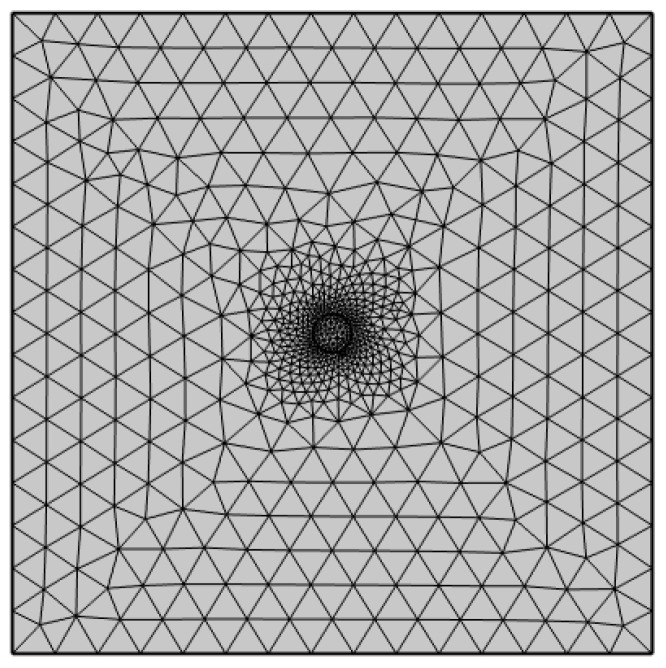
Non-isometric triangular mesh with variable density for the fabric equivalent model.

The quality of the mesh in a finite element model directly impacts the accuracy of computational results. Since this fabric equivalent model is a simple two-dimensional planar model, a non-isometric free triangular mesh division was performed for the simulation area, as shown in Figure 7. In the figure, the mesh density is increased in regions where the physical solution changes significantly (such as wetting the center region) and reduced in the edge region where the physical solution changes relatively insignificantly. This approach aims to balance computational efficiency with the attainment of high-precision solutions.

COMSOL Multiphysics finite element analysis was applied to transiently solve the above mathematical model. The output time step is set to 1 s, with a total of 60 iterative steps, and the tolerance selects the physical field control. Initially, the pressure values for each node were iteratively obtained, followed by calculating the flow rate for each node using Darcy’s law. The coupled solution provided the distribution of the moisture volume fraction across different parts of the fabric during the operation period. Finally, the temperature changes at each point, while drawing the isotherm of the fabric.

**Figure 8:**
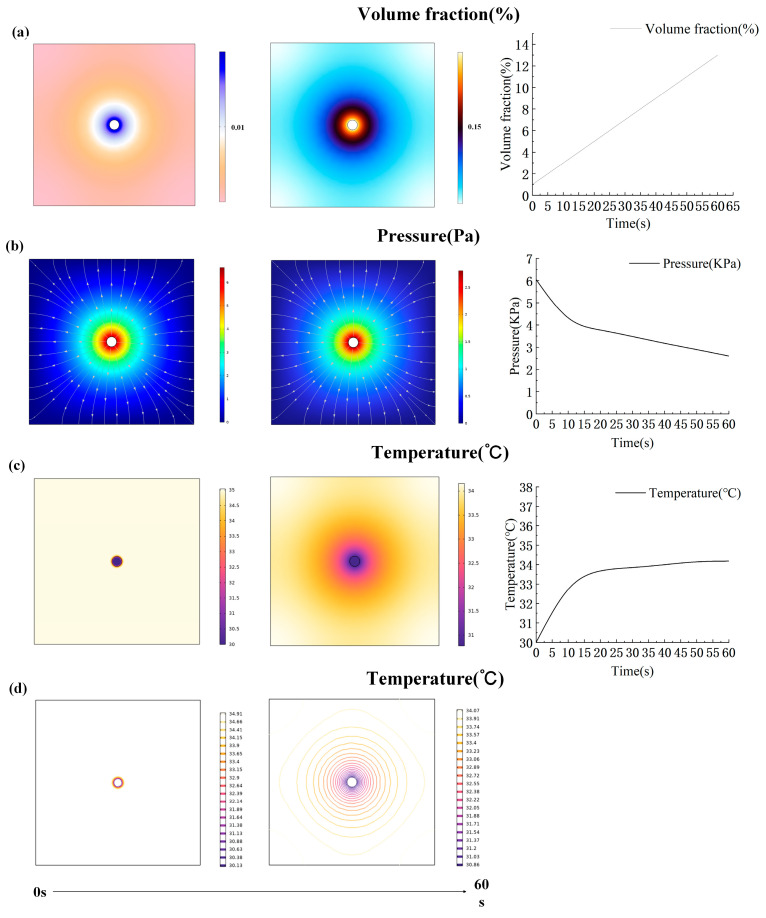
Simulation of thermal and moisture conduction in CP-2. (**a**) Moisture volume fraction change in fabric. (**b**) Moisture pressure change in fabric. (**c**) Fabric temperature change. (**d**) Fabric isotherm change.

**Figure 9:**
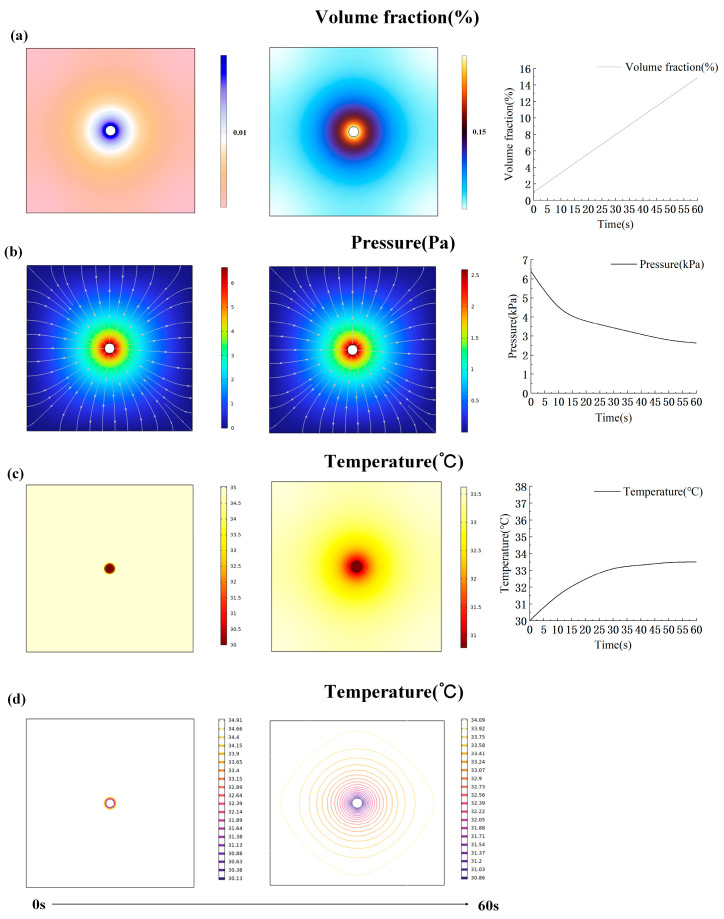
Simulation of thermal and moisture conduction in CP-1. (**a**) Moisture volume fraction change in fabric. (**b**) Moisture pressure change in fabric. (**c**) Fabric temperature change. (**d**) Fabric isotherm change.

The corresponding parameters were set and the simulation calculation was carried out. The transfer simulation process of moisture and heat for the fabric was studied. The results are shown in Figure 8 and Figure 9. The simulation results include the volume fraction and surface heat distribution. In the study of simulating the change in fabric moisture transfer, the volume fraction of moisture in the fabric gradually increased. This observation suggests that as water droplets infiltrate the fabric, moisture diffuses progressively across its surface, reaching a maximum volume fraction of 0.13 on the fabric’s surface (Figure 8a). Additionally, the simulation revealed a gradual decrease in moisture pressure within the fabric over time. Initially, as water droplets penetrate the fabric center, pressure peaks are observed. Subsequently, as moisture spreads, the contact area between the water droplets and fabric increases, leading to a gradual reduction in pressure (Figure 8b).

In the study of simulating the change in fabric heat, the fabric temperature was initially set to a constant 35 °C to mimic human body temperature. Upon water droplets landing and gradually seeping into the fabric, the core temperature of the fabric rapidly dropped to 30 °C. The overall temperature of the fabric increased as the water droplets gradually spread. As shown in Figure 8c, the temperature rises rapidly in the first 15 s, followed by a more gradual rise. Initially, upon water droplet impact, the isotherm exists in the center position of the fabric and its periphery, as shown in Figure 8d. However, during the process of simulation, the isotherm expanded gradually across the fabric surface. The temperature gradient decreased from the periphery towards the center of the fabric, with the density of the isotherm increasing correspondingly from the periphery towards the center. Compared to CP-2, CP-1 exhibits a significantly larger volume fraction. This indicates that a lower proportion of Coolmax reduces moisture wicking capability, making it more difficult for the central water droplet to spread. Consequently, the volume fraction in the central region increases. Regarding the temperature variation in the fabric, the CP-1 blend has smaller pores and retains less air, resulting in poorer insulation. When water is introduced, the surface temperature of the fabric drops rapidly, leading to a lower final surface temperature. This indicates that fabrics with a lower Coolmax content have better thermal conductivity compared to those with a higher Coolmax content (Figure 9).

**Figure 10:**
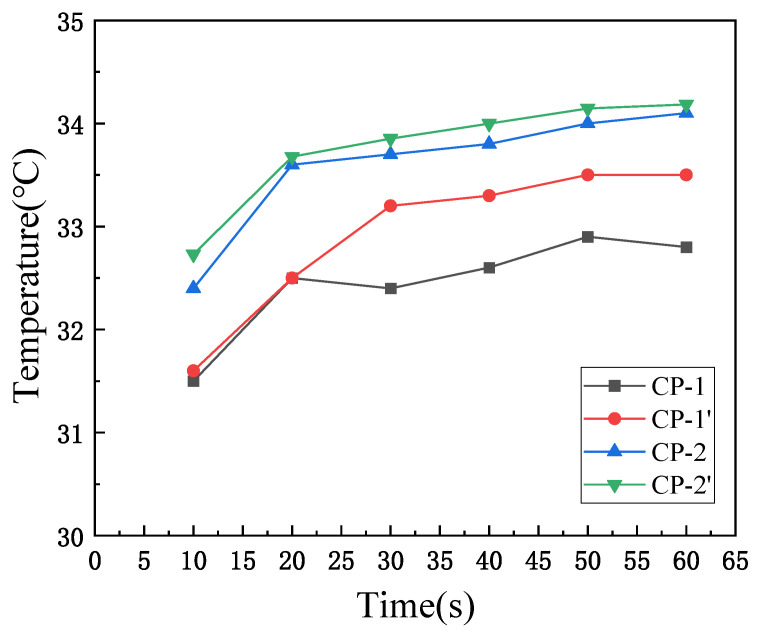
Comparison of simulated and actual results: CP-1 and CP-2 represent actual results; CP-1′ and CP-2′ represent simulated results.

Compared to the actual temperature change results in Figure 10, the overall trend of the simulated data is consistent with the experimental results. In practical scenarios, heat generated is directly dissipated into the environment, and the flow of air accelerates this heat loss. As a result, the simulated temperature rise of the fabric is higher than the corresponding experimental data. Since the CP-1 fabric contains less Coolmax, the number of fine grooves within the fabric structure is also reduced, leading to enhanced heat dissipation. Therefore, for CP-1, the simulated results are larger compared to the actual results, but the error does not exceed 2.3%. For CP-2, the simulated results are closer to the actual results, with an error not exceeding 0.5%.

## 4. Conclusions

In this study, weft-knitted fabrics were fabricated using Coolmax fiber with various concentrations to enhance the hygroscopic and perspiratory properties. The Coolmax/PP fabric has good stretchability, with a strain of 180.5%, and a high cumulative individual transfer capability that reached 691.6% with a water absorption rate of 50.2% per second. The trend of moisture and heat change in the fabric changes rapidly within 60 s, which reflects the good moisture and heat transfer performance of the knitted fabric. Furthermore, the heat–moisture transfer behavior was analyzed using mathematical modeling and visual simulation. The heating data from the thermal simulation are in close agreement with the experimental temperature rise. Overall, these findings provide a facile avenue for the rational design and effective construction of durable hydroscopic and sweat-discharging knitted fabrics, and the scaled-up preparation of multifunctional textiles.

## Figures and Tables

**Table 1:** Structural parameters of fabrics.

Fabric	CP-1		CP-2		CP-3	
Thickness/mm	0.72		0.61		0.71	
CV(%)	1.60		2.74		2.51	
Fabric Density/cm	Vertical	Horizontal	Vertical	Horizontal	Vertical	Horizontal
	25.76	25.23	25.33	25.66	25.67	25.33
CV(%)	2.82	3.71	4.22	2.07	1.45	5.58
Average surface wetting time (s)	6.39		5.14		4.4	
CV(%)	11.10		14.49		15.01	
Average water absorption rate (%/s)	47.5		49.1		50.2	
CV(%)	9.76		3.51		10.23	
Average maximum wetting radius (mm)	20		25		30	
CV(%)	10.1		9.1		7.5	
Average surface liquid water diffusion rate (mm/s)	3.2		3.4		3.5	
CV(%)	5.1		9.1		6.0	
Cumulative Individual Transfer Capability (%)	615.4		672.5		691.6	
CV(%)	4.4		3.9		6.7	

**Table 2:** Fabric model parameters.

Material	Density (Kg/m^3^)	Thermal Conductivity (W/(m*K))	Constant Pressure Heat Capacity (J/K*mol)	Porosity	Permeability (m^2^)
Coolmax/PP 60/40	224.10	0.06	0.30	0.75	0.08
Coolmax/PP 50/50	215.3	0.07	0.28	0.63	0.06

## Data Availability

The original contributions presented in the study are included in the article, further inquiries can be directed to the corresponding author.
